# Comparison between *Arabidopsis* and Rice for Main Pathways of K^+^ and Na^+^ Uptake by Roots

**DOI:** 10.3389/fpls.2016.00992

**Published:** 2016-07-05

**Authors:** Manuel Nieves-Cordones, Vicente Martínez, Begoña Benito, Francisco Rubio

**Affiliations:** ^1^Biochimie et Physiologie Moléculaire des Plantes, Institut de Biologie Intégrative des Plantes, UMR 5004 CNRS/UMR 0386 INRA/Montpellier SupAgro/Université Montpellier 2Montpellier, France; ^2^Departamento de Nutrición Vegetal, Centro de Edafología y Biología Aplicada del Segura – Consejo Superior de Investigaciones CientíficasMurcia, Spain; ^3^Centro de Biotecnología y Genómica de Plantas, Universidad Politécnica de MadridMadrid, Spain

**Keywords:** potassium, sodium, uptake, roots, *Arabidopsis*, rice

## Abstract

K^+^ is an essential macronutrient for plants. It is acquired by specific uptake systems located in roots. Although the concentrations of K^+^ in the soil solution are widely variable, K^+^ nutrition is secured by uptake systems that exhibit different affinities for K^+^. Two main systems have been described for root K^+^ uptake in several species: the high-affinity HAK5-like transporter and the inward-rectifier AKT1-like channel. Other unidentified systems may be also involved in root K^+^ uptake, although they only seem to operate when K^+^ is not limiting. The use of knock-out lines has allowed demonstrating their role in root K^+^ uptake in *Arabidopsis* and rice. Plant adaptation to the different K^+^ supplies relies on the finely tuned regulation of these systems. Low K^+^-induced transcriptional up-regulation of the genes encoding HAK5-like transporters occurs through a signal cascade that includes changes in the membrane potential of root cells and increases in ethylene and reactive oxygen species concentrations. Activation of AKT1 channels occurs through phosphorylation by the CIPK23/CBL1 complex. Recently, activation of the *Arabidopsis* HAK5 by the same complex has been reported, pointing to CIPK23/CBL as a central regulator of the plant’s adaptation to low K^+^. Na^+^ is not an essential plant nutrient but it may be beneficial for some plants. At low concentrations, Na^+^ improves growth, especially under K^+^ deficiency. Thus, high-affinity Na^+^ uptake systems have been described that belong to the HKT and HAK families of transporters. At high concentrations, typical of saline environments, Na^+^ accumulates in plant tissues at high concentrations, producing alterations that include toxicity, water deficit and K^+^ deficiency. Data concerning pathways for Na^+^ uptake into roots under saline conditions are still scarce, although several possibilities have been proposed. The apoplast is a significant pathway for Na^+^ uptake in rice grown under salinity conditions, but in other plant species different mechanisms involving non-selective cation channels or transporters are under discussion.

## Introduction

Given the constant increase in world population, high-yield crop production has become a necessity for agriculture. As that the nutrient sources of the land are limited, the input of nutrients by the addition of fertilizers ensures a continuous supply for plants, circumventing reductions in plant yield. The use of fertilizers has raised crop yield considerably, for example, from 50 to 80% of wheat and corn grain yield is attributable to nutrient fertilization (Stewart et al., 2005). However, this practice comes with high economic and environmental costs.

Potassium (K^+^) is an essential macronutrient that is required by plants to complete their life cycle (Taiz and Zeiger, 1991). K^+^ can make up to 10% of the total plant dry weight (Leigh and Wyn Jones, 1984) and fulfills important functions for metabolism, growth, and stress adaptation. Specifically, it acts as an enzyme activator, protein synthesis stabilizer, neutralization of protein negative charges, and it participates in cytoplasmic pH homeostasis as well (Marschner, 2012). An optimal K^+^ concentration in the cytosol of around 100 mM is required for the performing of the functions mentioned above (Wyn Jones and Pollard, 1983), and plant cells maintain the cytosolic K^+^ concentration around this value (Walker et al., 1996).

K^+^ constitutes about 2.9% of the earth’s crust but the concentration of K^+^ in the soil solution is highly variable, in the 10^-5^ to 10^-3^ M range (Barraclough, 1989; Marschner, 2012). Since roots are able to take up K^+^ at a higher rate than this cation can diffuse from the bulk soil solution, a K^+^ depletion zone near the root surface can be formed with K^+^ concentrations of just a few micromolar (Baldwin et al., 1973; Claassen and Jungk, 1982). More importantly, increasing areas of the world are currently described as being K^+^ deficient for agricultural practices (Mengel et al., 2001; Moody and Bell, 2006; Römheld and Kirkby, 2010; Kirkby and Schubert, 2013).

K^+^ deficiency has a negative impact on plant growth since cellular expansion and photosynthesis are severely affected under these conditions (Bednarz and Oosterhuis, 1999; Hafsi et al., 2014). This deficiency also correlates with a decrease in protein synthesis and subsequent decline in growth (Walker et al., 1996, 1998). K^+^ deficiency has been shown to inhibit lateral root development in *Arabidopsis* (Armengaud et al., 2004; Shin and Schachtman, 2004; Kellermeier et al., 2014) and in barley (Drew, 1975) and in the up-regulation of genes involved in K^+^ uptake (Ashley et al., 2006; Nieves-Cordones et al., 2014). In addition, K^+^-deficient plants are more sensitive to abiotic and biotic stresses such as drought, cold, salinity, or fungal attacks (Marschner, 2012; Zörb et al., 2014).

Sodium (Na^+^) is not an essential element for plants but, for some species it can be a beneficial element that stimulates growth (Wakeel et al., 2010, 2011; Kronzucker et al., 2013). In these cases, Na^+^ can be regarded as a functional nutrient (Subbarao et al., 2003), that can partly replace K^+^ in some functions such as osmotic adjustment of the large central vacuole, cell turgor regulation leading to cell enlargement, or long-distance transport of anions (Subbarao et al., 2003; Horie et al., 2007; Gattward et al., 2012; Battie-Laclau et al., 2013).

On the other hand, Na^+^ has been extensively associated to its negative impact on crop yield. Excess of Na^+^ salts in the soil results in both reduced soil water availability (due to the decrease in water potential) and ionic toxicity. When accumulated at high concentrations in the cytoplasm, Na^+^ results in deleterious effects on cell biology, e.g., on photosynthetic activity or on membrane integrity (due to displacement of membrane-bound Ca^2+^ ions) (Cramer et al., 1985). Thus, Na^+^ is usually compartmentalized outside the cytoplasm (Morgan et al., 2014), in vesicles such as the vacuole, where it is used as an osmoticum. Estimates of the area of salt-affected soils vary widely, ranging from 6 to 10% of the earth’s land area (Eynard et al., 2005; Munns and Tester, 2008). Importantly, 20% of irrigated lands are affected by secondary salinization, limiting agriculture worldwide.

In the present review, we summarize recent advances in the field of K^+^ and Na^+^ uptake in the plant root, with special attention to the transport systems and their regulation mechanisms. We believe that the studies performed on the model plant *Arabidopsis* and the results of recent research in crops such as rice suggest that the results obtained with model species cannot be fully extended to other plant species.

## K^+^ and Na^+^ Uptake By Roots: Kinetic Features and Sensitivity to Other Cations

K^+^ and Na^+^ can enter the root apoplast and diffuse toward inner cell layers (Sattelmacher et al., 1998). However, this pathway is interrupted by the endodermis, where the Casparian strip, which is impermeable to water and ions, is located (Schreiber et al., 1999; Tester and Leigh, 2001; Marschner, 2012; Geldner, 2013; Barberon and Geldner, 2014). To cross this impermeable barrier, nutrient ions enter the cytosol of a root peripheral cell either from the epidermis, cortex or endodermis and move from cell to cell (symplastic pathway) through plasmodesmata (Burch-Smith and Zambryski, 2012). Diffusion within the symplasm beyond the endodermic barrier allows nutrient ions to reach the stele, where they will initiate their travel toward the aerial parts within the xylem vessels (Lauchli, 1972).

It is worth noting that the Casparian strip may be absent in some places (Maathuis, 2014) allowing ions to reach the root stele and xylem vessels through the apoplastic pathway via bypass flow (Kronzucker and Britto, 2011). Since this flow is relatively low, most of the ions that reach the root xylem vessels are probably taken up across the plasma membrane of a root peripheral cell (Tester and Leigh, 2001). Thus, their entry into the root symplasm would have been mediated by membrane transport systems, channels, transporters or cotransporters. It should be noted that the bypass flow was observed in rice at Na^+^ external concentrations as low as 25 mM (Yeo et al., 1987) and it may contribute to salt stressing effects under high salinity by having an effect in shoot Na^+^ content (Yeo et al., 1987; Faiyue et al., 2012; Maathuis, 2014). Na^+^ bypass flow has been described in other species, besides rice, such as mangroves (Krishnamurthy et al., 2014), maize, and broad bean (Peterson et al., 1981), but not in *Arabidopsis* (Essah et al., 2003).

More than 60 years ago, through the application of the concept of enzyme kinetics for the study of root K^+^ absorption (Epstein and Hagen, 1952), Epstein et al. (1963) suggested that at least two transport systems were involved in root K^+^ uptake: a high-affinity system that operates at low external concentrations and a low-affinity system at higher concentrations. A similar scheme was also described for Na^+^ uptake (Rains and Epstein, 1967a). This biphasic behavior has since been observed in many plant species, with some exceptions. Maize, for example, shows a linear, non-saturating response to K^+^ in the low-affinity range (Kochian and Lucas, 1982). More recently, it has been shown that this linear response is dominated by the apoplastic movement of K^+^, and the “true” transmembrane flux saturates at modest rates (Coskun et al., 2016). In the high-affinity range of concentrations, K^+^ uptake is an active process that takes place against the K^+^ electrochemical potential, most likely by a K^+^/H^+^ symport, whilst the low-affinity uptake can take place by passive transport through inwardly rectifying K^+^ channels (Maathuis and Sanders, 1996a; Maathuis et al., 1997; Rodríguez-Navarro, 2000). It should also be noted that the limits in K^+^ concentrations for the operation of a symporter or a channel depend on the plasma membrane potential and the cytoplasmic pH and K^+^ concentrations. Thus, assuming, for example, that a cytoplasmic K^+^ concentration of 100 mM and a membrane potential of -240 mV exists, K^+^ uptake could take place through a channel from an external K^+^ concentration as low as 10 μM, which falls within the high-affinity system described by Epstein et al. (1963), Hirsch et al. (1998) and Spalding et al. (1999). High-affinity K^+^ uptake becomes apparent when K^+^ tissue concentrations decrease due to K^+^ starvation (Glass, 1976; Kochian and Lucas, 1982; Siddiqi and Glass, 1986; Martínez-Cordero et al., 2005). Providing NH_4_^+^ to the nutrient solution used to grow the plants, has a large influence on the NH_4_^+^ sensitivity of high-affinity K^+^ uptake. In some species such as barley (Santa-María et al., 2000), pepper (Martínez-Cordero et al., 2005), or *Arabidopsis* (Rubio et al., 2008), the presence of NH_4_^+^ in the growth solution induced an NH_4_^+^-insensitive high-affinity K^+^ uptake component. In others, such as tomato (Nieves-Cordones et al., 2007), high-affinity K^+^ uptake was dominated by an NH_4_^+^-sensitive component, irrespectively of the presence or the absence of NH_4_^+^ in the growth solution. By contrast, both Na^+^ (Martínez-Cordero et al., 2005; Kronzucker et al., 2006, 2008; Nieves-Cordones et al., 2007, 2010; Alemán et al., 2009; Cheng et al., 2015) and Cs^+^ (Rubio et al., 2000; White and Broadley, 2000; Qi et al., 2008) usually inhibit high-affinity K^+^ uptake.

Regarding low-affinity K^+^ transport, the role of TEA^+^, Cs^+^, and Ba^2+^, in blocking animal and plant K^+^ channels is well known, as these inhibit low-affinity K^+^ uptake, supporting the idea of the channel-mediated nature of this transport (Ketchum and Poole, 1991; Blatt, 1992; Hille, 1992; Very and Sentenac, 2002; Hoopen et al., 2010; Coskun et al., 2013). Unlike high-affinity K^+^ uptake, low-affinity K^+^ uptake is not down-regulated at high external K^+^ (Maathuis and Sanders, 1996b; Szczerba et al., 2006) and it is NH_4_^+^-insensitive (Spalding et al., 1999; Santa-María et al., 2000; Kronzucker et al., 2003; Szczerba et al., 2006). Na^+^ suppresses K^+^ uptake both in the low- and the high-affinity ranges, with low-affinity K^+^ uptake being more sensitive to this inhibition (Epstein et al., 1963; Rains and Epstein, 1967a; Kronzucker et al., 2006, 2008). It is worth noting that K^+^ uptake seems to be insensitive to Ca^2+^ in barley and *Arabidopsis* (Cramer et al., 1989; Caballero et al., 2012; Coskun et al., 2013).

As for Na^+^ uptake, it has been shown that root high-affinity Na^+^ uptake is significant when plants are starved of K^+^ (Garciadeblas et al., 2003; Haro et al., 2010). Under these conditions, Na^+^ is able to partially replace K^+^ and support plant growth (Maathuis and Sanders, 1993; Garciadeblas et al., 2003; Horie et al., 2007; Wakeel et al., 2011; Wakeel, 2013). High-affinity Na^+^ uptake has been shown to be sensitive to K^+^ and Ca^2+^ in barley (Rains and Epstein, 1967b) and to K^+^ and Ba^2+^ in rice (Garciadeblas et al., 2003). By contrast, low-affinity Na^+^ uptake seems to be insensitive to Ca^2+^ and K^+^ in rice (Malagoli et al., 2008) while it is sensitive to Ca^2+^ in *Arabidopsis*, barley, and wheat (Cramer et al., 1987, 1989; Essah et al., 2003; D’Onofrio et al., 2005). Na^+^ uptake can be reduced by the application of K^+^, and such reduction can alleviate salt stress effects to some extent (Wakeel, 2013). For example, a reduction in tissue Na^+^ content due to increased K^+^ application was observed in strawberry or in *Jatropha curcas* (Khayyat et al., 2009; Rodrigues et al., 2012).

## Identification of K^+^ Transport Systems in Plants

The molecular approaches developed in the last 25 years have led to the characterization of many K^+^ and Na^+^ transport systems in plants. The first K^+^ uptake system identified in plants, AKT1, was isolated by complementing a K^+^-uptake deficient yeast strain with an *Arabidopsis* cDNA library (Sentenac et al., 1992). Sequence analysis and heterologous expression in Sf9-insect cells showed that the AKT1 cDNA encoded an inward-rectifier K^+^ channel belonging to the Shaker family (Gaymard et al., 1996). The gene encoding this channel showed specific constitutive expression in epidermal root cells (Lagarde et al., 1996) and AKT1 was proposed as the system mediating low-affinity K^+^ uptake in the roots.

Later, a PCR-based approach led to the identification of a cDNA from barley, HvHAK1, that mediated high-affinity K^+^ uptake in yeast (Santa-María et al., 1997). Specific expression of its mRNA in K^+^-starved roots and its kinetic properties in yeast prompted researchers to propose it as the system mediating the high-affinity K^+^ uptake observed in barley roots (Epstein et al., 1963). Subsequent studies led to the identification of an *Arabidopsis* homolog of HvHAK1, that was named AtHAK5 (Rubio et al., 2000).

In addition to AKT1 and AtHAK5-like transporters, other K^+^ uptake systems could be involved in root K^+^ uptake and in trans-membrane K^+^ movements in other plant organs. Moreover, the sequence of whole genomes of plants evidenced the existence of large gene families encoding putative K^+^ transport systems (Grabov, 2007; Véry et al., 2014; Nieves-Cordones et al., 2016).

### Initial Characterizations in *Arabidopsis*

The studies on heterologous systems and on gene expression patterns for AKT1 and AtHAK5 produced data suggesting that these two systems played important roles in K^+^ acquisition by the root. However, a demonstration for the proposed roles was only possible when *Arabidopsis* knock-out mutants for these two genes became available (Spalding et al., 1999; Gierth et al., 2005; Rubio et al., 2008, 2010; Pyo et al., 2010). The studies showed that while *Arabidopsis* wild-type (WT) and *akt1* plants could deplete external K^+^ (Rb^+^) to values around 1 μM, the *athak5* plants did not diminish its concentration below 30 μM. In agreement with this, reduced growth was observed in *athak5* plants grown in the presence of 1 μM K^+^ (Qi et al., 2008) or 10 μM K^+^ (Pyo et al., 2010; Ragel et al., 2015). Moreover, K^+^ (Rb^+^) uptake in *athak5* plants was completely inhibited by the presence of Ba^2+^ in the external solution. By contrast, a complete inhibition of K^+^ (Rb^+^) depletion was observed in the presence of NH_4_^+^ in the *akt1* line. An *athak5 akt1* double mutant did not show K^+^ (Rb^+^) uptake at external concentrations below 50 μM, and it could only promote K^+^ (Rb^+^) uptake at concentrations higher than 100 μM. All these results demonstrated that in *Arabidopsis* plants, AtHAK5 was the only system mediating K^+^ uptake at concentrations below 10 μM and that this system was inhibited by NH_4_^+^. At concentrations between 10 and 200 μM, both AtHAK5 and AKT1 contributed to K^+^ uptake, defining AKT1 as a Ba^2+^-sensitive component of K^+^ uptake. Above 500 μM AtHAK5 contribution was negligible as the *AtHAK5* gene was repressed at this external K^+^ concentration, and AKT1 role became more relevant. Unidentified systems could compensate for the lack of AKT1 since the *akt1* and the *athak5 akt1* lines grew at similar rates than the WT line when the external K^+^ concentration was sufficiently high (∼10 mM).

The studies described for *Arabidopsis* allowed for depicting a model for the contribution of AtHAK5 and AKT1 to root K^+^ uptake (Alemán et al., 2011). They also allowed for extending the studies to species where knock-out mutants were not available, through the use of NH_4_^+^ and Ba^2+^ as specific inhibitors of HAK5 and AKT1, respectively. Thus, it was shown that in tomato and pepper plants grown in the absence of NH_4_^+^, an NH_4_^+^-sensitive component, probably mediated by SlHAK5 (formerly LeHAK5) and CaHAK1, respectively, dominated K^+^ uptake from concentrations corresponding to the high-affinity component (inhibition of K^+^ uptake by NH_4_^+^ was close to 80% in tomato, for example; Martínez-Cordero et al., 2005; Nieves-Cordones et al., 2007). These results contrast with those obtained for *Arabidopsis*, where both AtHAK5 and AKT1 contribute to K^+^ uptake within the range of the K^+^ concentration assigned to the high-affinity component, as demonstrated by the use of single (Rubio et al., 2008), and double KO mutants (Rubio et al., 2010) in AtHAK5 and AKT1. Therefore, it can be concluded that the *Arabidopsis* model cannot be completely extended to other plant species, crops included, and highlights the need for the characterization of knock-out lines in each particular species to address the relevance of the different K^+^ transport systems. In addition, the availability of whole genomes for many plant species revealed some differences with respect to *Arabidopsis*. In *Arabidopsis*, AtHAK5 is the only member that belongs to the cluster Ia of HAK transporters, which are involved in high-affinity K^+^ uptake in roots (Nieves-Cordones et al., 2016). In tomato, a highly homologous gene to *SlHAK5* is located just 2580 bp downstream from it in the tomato genome (Fernandez-Pozo et al., 2015). In rice, the *OsHAK21* gene, also belonging to cluster Ia is induced by salinity, something that has not been described in other species for members of this cluster. This transporter has been linked to rice tolerance to salinity through the maintenance of Na^+^/K^+^ homeostasis, although the physiological mechanism remains unclear (Shen et al., 2015).

### Rice Transport Systems Contributing to Root K^+^ Uptake

The recent characterization of T-DNA insertion rice lines knocked-out for K^+^ uptake systems such as OsHAK1, OsHAK5, and OsAKT1, has importantly contributed to increase our understanding of the relative contribution of such systems to K^+^ uptake in a species different from *Arabidopsis*, which is of great importance in agriculture.

A T-DNA insertion mutant with OsAKT1 knocked-out (Golldack et al., 2003) showed reduced growth and decreased root and shoot K^+^ concentrations when grown in the presence of 1 and 0.1 mM K^+^ (Li et al., 2014). K^+^ flux experiments and electrophysiological approaches showed an impairment of K^+^ uptake in the *osakt1* line. Strong expression of *OsAKT1* was detected in epidermal root cells, but it was also found in cortex, endodermis, and vascular bundles, suggesting a direct or indirect role in K^+^ translocation. In addition, slight expression was detected in shoots.

Knock-out mutants of the gene encoding the high-affinity K^+^ transporter OsHAK1 (Bañuelos et al., 2002) were also characterized. The studies with *oshak1* lines showed that OsHAK1 contributed about 50–55% of high-affinity K^+^ uptake in the range of 0.05–0.1 mM external K^+^ and about 30% of K^+^ uptake at 1 mM external K^+^ (Chen et al., 2015). Root and shoot growth, cell size and internal K^+^ concentrations were reduced in the *oshak1* mutant at both 0.1 and 1 mM K^+^ and this deficient phenotype could not be rescued at high external K^+^ (5 mM K^+^). Transcripts of *OsHAK1* are preferentially accumulated in roots of K^+^-starved plants, as it is observed with *AtHAK5* in *Arabidopsis*. In addition, *OsHAK1* is strongly expressed at the xylem parenchyma and phloem of root vascular tissues, shoot meristems and vascular bundles of leaf sheaths. Moreover, *oshak1* plants show reduced K^+^ translocation from root to shoot. In addition, the *osakt1* and *oshak1* lines were inhibited throughout development, showing delayed grain filling and reduced grain yield, suggesting that they may play an important role in rice productivity.

OsHAK5 was isolated and characterized as a high-affinity transport system in heterologous systems (Horie et al., 2011). Expression studies showed that OsHAK5 localized to the plasma membrane and that under normal K^+^ supply its transcripts were detected in root, root–shoot junction and leaf sheath. K^+^ starvation enhanced its expression in root epidermis, parenchyma of stele tissue, primordials of lateral roots, mesophyll, and parenchyma cells of the vascular bundle. The expression pattern of *OsHAK5* supported its role in K^+^ uptake, but also in K^+^ distribution between roots and shoots, especially at low external K^+^. The lower accumulation of K^+^ in roots of overexpressing lines and the higher K^+^ accumulation in the knock-out lines, grown in low K^+^, supported this idea (Yang et al., 2014). These authors suggested that OsHAK5 may mediate K^+^ accumulation in xylem parenchyma cells to enable K^+^ channels to release K^+^ efficiently into the xylem sap. A role for OsHAK5 in K^+^ signaling is also proposed, as K^+^ in the phloem may act as a signal to convey the shoot demand of K^+^ and K^+^ xylem loading (Engels and Marschner, 1992), and *OsHAK5* is abundantly expressed in phloem tissue. A role for the AKT2 channel in K^+^ signaling by modulating K^+^ in the phloem has been also proposed (Deeken et al., 2002) and AKT2 has been recently suggested as a pathway for Na^+^ entry into the roots (Salvador-Recatala, 2016). Since AKT2-mediated K^+^ transport is Ca^2+^-sensitive (Latz et al., 2007), interesting interactions between salt stress, Ca^2+^, and AKT2 may emerge.

The differential abundance of *OsHAK1*, *OsHAK5*, and *OsAKT1* transcripts suggests that they play non-redundant functions. *OsHAK1* and *OsAKT1* are highly expressed in all cell types of roots and at low levels in shoots. However, while the *OsHAK1* gene was induced 8- to 12-fold by K^+^ starvation (Chen et al., 2015), *OsAKT1* expression was not affected by the external K^+^ concentrations (Li et al., 2014). By contrast, *OsHAK5* was less expressed in roots and strongly in shoots (Yang et al., 2014). It has been proposed that both OsHAK1 and OsAKT1 contribute to K^+^ acquisition at low and high external concentrations. At low K^+^, OsHAK1 dominates high-affinity K^+^ uptake over OsAKT1 and OsHAK5. By contrast, OsHAK5 dominates K^+^ transport from root to shoots (Yang et al., 2014).

The studies described above suggest that OsHAK1, OsHAK5, and OsAKT1 are involved in K^+^ uptake at low and high concentrations as well as in K^+^ translocation from root to shoot. Contribution to K^+^ uptake over a wide range of K^+^ concentrations by a unique system has been demonstrated for the *Arabidopsis* AKT1 (Alemán et al., 2011). For transporters of the HAK family, the overexpression of AtKUP1 in *Arabidopsis* suspension cells produced enhanced K^+^ uptake at micromolar and millimolar K^+^ concentrations (Kim et al., 1998). The rice studies reviewed here suggest that OsHAK1 and OsHAK5 may contribute to K^+^ uptake at both low and high concentrations, which would explain why the growth of *oshak1* and *oshak5* lines is not rescued at high external K^+^. However, the idea of dual affinity for HAK transporters should be taken with caution. It is possible that, in addition to K^+^ uptake, the knock-out lines are affected in other processes. In fact, Yang et al. (2014) highlight the role of some HAK/KUP/KT transporters in auxin distribution, irrespective of K^+^ supply. At this stage, it cannot be ruled out that OsHAK1 and OsHAK5 play a part in this process.

### Role of HAK Transporters in K^+^/Na^+^ Homeostasis under NaCl Stress

K^+^/Na^+^ homeostasis has been shown to be crucial for tolerance of plants to salinity (Maathuis and Amtmann, 1999). Maintaining K^+^ uptake rates at high external Na^+^ is crucial for K^+^/Na^+^ homeostasis and salt tolerance (Munns and Tester, 2008; Cuin et al., 2012; Cheng et al., 2015). However, in the presence of high Na^+^ concentrations, the low-K^+^ induction of genes encoding high-affinity K^+^ transporters is not observed (Nieves-Cordones et al., 2008, 2010). In addition, under salinity, K^+^ transport through high-affinity HAK transporters is competitively inhibited (Santa-María et al., 1997; Rubio et al., 2000). In fact, OsHAK1 has been defined as the Na^+^-sensitive high-affinity K^+^ pathway in rice (Chen et al., 2015). Nonetheless, studies in low-K^+^-grown *Arabidopsis* and rice plants showed that AtHAK5 and OsHAK1 function was still pivotal in maintaining K^+^ uptake and plant growth in the presence of high Na^+^ (Nieves-Cordones et al., 2010; Chen et al., 2015). In addition to OsHAK1, OsHAK5 may also play a role in salt tolerance, as high salt transiently enhances *OsHAK5* expression (Yang et al., 2014) and the transporter mediates Na^+^-insensitive K^+^ uptake (Horie et al., 2011). In the presence of salinity, OsHAK5-overexpressing lines accumulated more K^+^ in shoots and showed enhanced growth as compared to WT. In contrast, *oshak5* lines accumulated more Na^+^ in shoots and grew less than WT plants (Yang et al., 2014). The authors propose that the higher accumulation of Na^+^ in shoots in *oshak5* may be due to a hyperpolarized membrane potential of mesophyll cells in knock-out mutants that would favor Na^+^ accumulation in shoots. Therefore, the K^+^ transport systems that contribute to maintaining a depolarized membrane potential of mesophyll cells to evade excessive Na^+^ accumulation under salinity may play a role in salt tolerance. The function of another rice HAK transporter, OsHAK21, seems to be important for salt tolerance (Shen et al., 2015). The gene encoding this transporter is enhanced by salinity and the protein is localized to the plasma membrane of xylem parenchyma and endodermal cells (putative passage cells). It has been shown that the knock-out *oshak21* line is more salt sensitive because of a higher and a lower accumulation of Na^+^ and K^+^ respectively, which points to OsHAK21 as a key transporter needed for the maintenance of Na^+^/K^+^ homeostasis in rice under salt stress.

### Other Systems That May Be Involved in Root K^+^ Uptake

Recently, a T-DNA insertion mutant in the *Arabidopsis* KUP7, which belongs to cluster V of the HAK family (Nieves-Cordones et al., 2016) has been characterized (Han et al., 2016). The results showed that the *kup7* line was sensitive to low K^+^ (<100 μM), showing lower internal K^+^ concentrations. It could be rescued by higher K^+^ concentrations or by complementing the mutant with the WT gene. The *KUP7* gene was ubiquitously expressed in many organs and the KUP7 protein was localized to the plasma membrane. KUP7 could complement a yeast strain deficient in K^+^ uptake. K^+^ transport studies showed that KUP7 was involved in root K^+^ uptake and K^+^ translocation to the shoot. It seemed to operate at higher concentrations than AtHAK5, and may be an alternative system involved in K^+^ uptake in the *athak5 akt1 Arabidopsis* line (Caballero et al., 2012). The observed effect on K^+^ translocation may be an indirect effect of a reduced uptake. However, Han et al. (2016) speculated that KUP7 may mediate K^+^ release into the xylem sap. It should be noted that besides K^+^ uptake, some HAK transporters have been shown to mediate K^+^ efflux (Bañuelos et al., 2002; Garciadeblas et al., 2002; Osakabe et al., 2013). Thus, K^+^ release into the xylem sap could take place through this type of transporters, if the electrochemical potential for K^+^ allows for this movement. It is well known that K^+^ loading of the xylem is mainly mediated by SKOR channels and the possible specific contribution of HAK transporters to K^+^ loading is yet to be determined.

**FIGURE 1:**
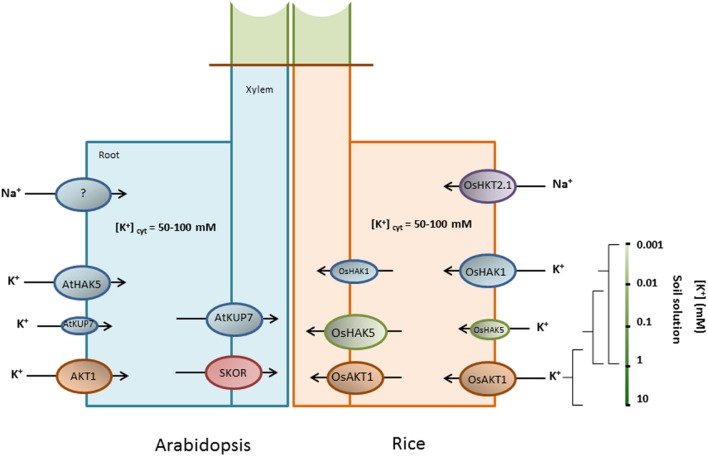
**Schematic comparison of the systems involved in K^+^ and Na^+^ movements in *Arabidopsis* and rice roots.** The availability of knock-out mutants in *Arabidopsis* and rice plants for specific transport systems has allowed for elucidating their roles in K^+^ transport. The figure shows the predicted function of each system for which knock-out mutants have been studied. AtHAK5 and AKT1 are the main systems for K^+^ uptake in *Arabidopsis* plants. In addition, a member of the KT/HAK/KUP family, AtKUP7, seems to also be involved in K^+^ uptake. K^+^ release into the xylem is mainly mediated by SKOR and also partially by AtKUP7. In rice, AtHAK5 and AKT1 functions are fulfilled by the rice homologs OsHAK1 and OsAKT1. An additional system, OsHAK5, partially contributes to high-affinity K^+^ uptake, but at higher concentrations than OsHAK1. These three rice systems may directly or indirectly facilitate K^+^ release into the xylem, with the contribution by OsHAK5 to K^+^ release into the xylem being more relevant. It is not clear if such contribution is a direct (by mediating K^+^ efflux into xylem vessels) or indirect (by favoring K^+^ accumulation in endodermal cells) outcome of the aforementioned transporters. Regarding Na^+^ uptake, the genetic identity of Na^+^ uptake systems in *Arabidopsis* remains to be elucidated. GLRs, CNGCs or other non-selective cation channels could be involved. In rice, OsHKT2;1 has been shown to mediate Na^+^ uptake during K^+^ deficiency. In the presence of K^+^ or high external Na^+^ concentrations other unknown systems should take part in Na^+^ uptake.

All of the above described results can be summarized into two different models for the K^+^ uptake systems in *Arabidopsis* and rice roots, shown in **Figure 1** and **Table 1.**

**Table 1:** Summary for K^+^ and Na^+^ uptake features observed in *Arabidopsis* and rice roots.

Species	Cation	Type of uptake^†^	*K*_m_ (μM)	Sensitivity	Transport systems involved	Reference
*Arabidopsis*	K^+^	High-affinity	24^∗^	(-) NH_4_^+^, Ba^+2^, Cs^+^ (=) Ca^+2^	AtHAK5, AKT1	Spalding et al., 1999; Gierth et al., 2005; Rubio et al., 2008; Coskun et al., 2013
		Low-affinity	3,991^∗^	(-) Ba^+2^, Cs^+^, TEA, La^+3^, Na^+^ (=) NH_4_^+^, Ca^+2^	AKT1	Spalding et al., 1999; Gierth et al., 2005; Caballero et al., 2012
	
	Na^+^	High-affinity	n.d.	n.d.	–	–
		Low-affinity	Linear response	(-) Ca^+2^, Ba^+2^, cyclic-nucleotides (+) La^+3^, GABA (=) TEA, Cs^+^	–	Maathuis and Sanders, 2001; Essah et al., 2003

Rice	K^+^	High-affinity	11–18	(-) NH_4_^+^	OsHAK1, OsHAK5, OsAKT1	Bañuelos et al., 2002; Li et al., 2014; Yang et al., 2014; Chen et al., 2015
		Low-affinity	n.d.	n.d.	OsHAK1, OsAKT1	Li et al., 2014; Chen et al., 2015
	
	Na^+^	High-affinity	60 or 477–655^¶^	(-) K^+^, Ba^+2^	OsHKT2;1	Garciadeblas et al., 2003; Horie et al., 2007; Haro et al., 2010
		Low-affinity	n.d.	(=) K^+^, Ca^2+^	OsHKT2;1	Horie et al., 2007; Malagoli et al., 2008


*^†^High-affinity uptake takes into account cation uptake observed at external concentrations <0.5 mM while the low-affinity one does so at >0.5 mM. ^∗^Obtained with Rb^+^ as an analog for K^+^. (+), (-), and (=) stand for activation, inhibition, or no effect on cation uptake, respectively. n.d., not determined. ^¶^ Only one component was identified in Horie et al. (2007) for external Na^+^ concentrations up to 5 mM.*

## Regulation of K^+^ Uptake

In general terms, the genes encoding HAK transporters that mediate high-affinity K^+^ uptake in roots are strongly induced by K^+^ deprivation, whereas the genes encoding AKT1 channels are not. Several elements in the signal transduction cascade that results in the activation of HAK transcription have been identified. One of the first events when a root faces K^+^ deprivation is the hyperpolarization of the cell’s plasma membrane (Amtmann and Blatt, 2009). A positive correlation has been found between the membrane potential and the expression levels of *SlHAK5* and *AtHAK5*, independent of the root’s K^+^ concentration (Nieves-Cordones et al., 2008; Rubio et al., 2014). Thus, it has been proposed that the hyperpolarization of the membrane potential may be the first element in the low-K^+^ signal cascade. The mechanisms linking membrane potential and gene expression are unknown but, changes in cytoplasmic Ca^2+^ derived from the activity of hyperpolarization-activated Ca^2+^ channels, could provide a connecting mechanism (Véry and Davies, 2000). Increases in ethylene (Jung et al., 2009) and reactive oxygen species (ROS; Shin and Schachtman, 2004; Hernandez et al., 2012) are also involved, probably acting following the hyperpolarization of the membrane potential. Other hormones such as jasmonic acid (Armengaud et al., 2004, 2010; Takehisa et al., 2013; Schachtman, 2015) and cytokinins (Nam et al., 2012) may also play a role in K^+^ signaling. At the end of the cascade, transcription factors such as DDF2, JLO, bHLH121, TFII_A for *Arabidopsis AtHAK5* (Kim et al., 2012; Hong et al., 2013), bind the gene’s promoter to activate its expression.

Interestingly, some environmental conditions such as the lack of N, P, or S, that hyperpolarize root cell membrane potential (Rubio et al., 2014) and elevate ROS levels (Shin et al., 2005), also activate the transcription of *AtHAK5*-type genes. However, under such conditions, no HAK-mediated uptake is observed, suggesting a post-transcriptional regulation for these HAK transporters that is elicited specifically by K^+^ starvation (Rubio et al., 2014). Recently, it has been shown that the *Arabidopsis* AtHAK5 transporter is activated by complexes that contain the CIPK23 kinase and CBL1, CBL8, CBL9, or CBL10 Ca^2+^ sensors. AtHAK5 phosphorylation by CIPK23 leads to increases in the maximal rate of transport (*V*_max_) and the affinity for K^+^ transport (Ragel et al., 2015). It can be assumed that a specific low-K^+^-induced Ca^2+^ signal is registered by the CBL Ca^2+^ sensor, that in turn promotes phosphorylation of the transporter by CIPK23.

As a voltage-dependent inward-rectifier K^+^ channel, AKT1 activity is regulated by the membrane potential (Gaymard et al., 1996; Xu et al., 2006). In addition, its activity is modulated by interaction with other proteins. AKT1 forms tetrameric channels by interacting with the AtKC1 subunit (Daram et al., 1997; Pilot et al., 2003; Duby et al., 2008; Geiger et al., 2009). Upon interaction with AtKC1 in root cells, the activation potential for AKT1-containing channels becomes more negative, in comparison with AKT1 homotetramers (Reintanz et al., 2002; Wang et al., 2010). Moreover, AtKC1 interacts, in turn, with the SNARE proteins SYP121 (Honsbein et al., 2009) and VAMP721 (Zhang et al., 2015). SYP121 was shown to activate K^+^ uptake through AKT1/AtKC1 channels while VAMP721 negatively regulated this process. Phosphorylation also plays a role in AKT1 regulation. AKT1 activity is enhanced at low K^+^ by the same regulators as AtHAK5, i.e., the CIPK23/CBL1/9 complex, supporting the putative role of Ca^2+^ as a secondary messenger involved in low-K^+^ signaling (Li et al., 2006; Xu et al., 2006). Inactivation of the channel is achieved by the AIP1 phosphatase (Luan, 2009). It is worth to note that CBL proteins can also modify AKT1 activity in absence of CIPKs, as it is the case of CBL10 which is a negative regulator of AKT1 (Ren et al., 2013). Recently it has been shown that CIPK23 and AtKC1 act synergistically and balance K^+^ uptake/leakage to modulate AKT1-mediated responses of *Arabidopsis* to low K^+^ (Wang et al., 2016). Finally, nitric oxide has recently been shown to lower AKT1 activity by modulating vitamin B6 biosynthesis, constituting a new mechanism for the regulation of K^+^ uptake (Xia et al., 2014).

**FIGURE 2:**
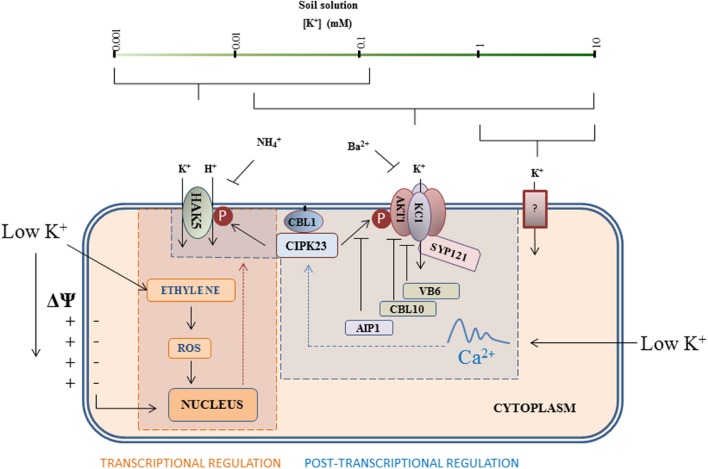
**Main pathways for root K^+^ uptake and their regulatory mechanisms.** HAK5-type transporters are high-affinity K^+^ transporters involved in K^+^ uptake at very low concentrations. When the external K^+^ concentration increases, the inward-rectifier K^+^ channel AKT1 together with HAK5, contributes to K^+^ uptake. At K^+^ concentrations above 200 μM, HAK5 is not present and AKT1 is the main system for low-affinity K^+^ uptake. At very high concentrations, other unknown systems can secure K^+^ supply if AKT1 is not functional. The HAK5 and AKT1 uptake systems are subjected to finely tuned regulation. At low K^+^ concentrations, a hyperpolarization of the plasma membrane induces *HAK5* transcription. The signal cascade of HAK5 regulation is dependent on ethylene and ROS production. In addition, low external K^+^ likely produces a specific cytoplasmic Ca^2+^ signal that is registered by the Ca^2+^ sensor CBL1, which induces CIPK23 recruitment to the plasma membrane resulting in the phosphorylation and subsequent activation of HAK5 and AKT1. The channel activity is downregulated through dephosphorylation by the AIP1 phosphatase, and interaction with CBL10 and vitamin B6. Other subunits such as KC1 and the SNARE protein SYP121 also cooperate in AKT1 regulation. It can be concluded that whereas HAK5 is subjected to transcriptional and post-transcriptional regulation, K^+^ uptake through AKT1 is mainly regulated post-transcriptionally. The CIPK23/CBL1 complex emerges as a key regulator of K^+^ nutrition.

The current model for the regulation of these two main systems involved in K^+^ uptake, i.e., AtHAK5 and AKT1, is depicted in **Figure 2.**

It is worth mentioning that CIPK23/CBL is also involved in the regulation of NO_3_^-^ uptake (Ho et al., 2009; Tsay et al., 2011; Léran et al., 2015), and that K^+^ starvation significantly reduced the NO_3_^-^ concentrations in tomato plants (Rubio et al., 2014) and induced several genes for NO_3_^-^ uptake (Armengaud et al., 2004). This indicates that a cross-regulation between K^+^ and NO_3_^-^ nutrition exists and that the CIPK23/CBL complex may constitute one of the key elements for such regulation.

## Uptake of Na^+^ and Cell Mechanisms Involved

As for the identities of genes involved in Na^+^ uptake from external solutions, the scenario is far less clear than that for K^+^. With respect to high-affinity Na^+^ uptake, despite this activity being widely observed in roots from several species (Garciadeblas et al., 2003; Haro et al., 2010), only a few transport systems belonging to the HKT and HAK transporter families have been shown to take up Na^+^ within this range of concentrations. High-affinity K^+^ transporters (HKT) are related to fungal and bacterial K^+^ transporters from the Trk/Ktr families (Corratgé-Faillie et al., 2010). In plants, however, HKT transporters display varying Na^+^/K^+^ permeabilities. Phylogenetic and functional analyses have led to the identification of two HKT subfamilies (Platten et al., 2006): subfamily I, present in both monocotyledonous and dicotyledonous species, and subfamily II, identified only in monocotyledonous species so far. Subfamily II HKT transporters are expected to be all K^+^-permeable and can operate as Na^+^/K^+^ symporters (Rubio et al., 1995; Jabnoune et al., 2009; Yao et al., 2010; Oomen et al., 2012) or K^+^-selective uniporters (Horie et al., 2011; Sassi et al., 2012) when heterologously expressed in yeast and/or *Xenopus* oocytes. Subfamily I HKT transporters are Na^+^-selective in *Arabidopsis* and rice and are mostly involved in Na^+^ recirculation through vascular tissues (Maathuis, 2014; Véry et al., 2014), thus falling beyond the scope of the present review. In the rice cultivar Nipponbare, OsHKT2;1 provides a major pathway for root high-affinity Na^+^ uptake that supports plant growth under limiting K^+^ supply (Garciadeblas et al., 2003; Horie et al., 2007; **Figure 1**). Plants lacking a functional *OsHKT2;1* gene have shown reduced growth and lower Na^+^ content when starved of K^+^ in the presence of 0.5 mM Na^+^, and under such conditions Na^+^ can partially compensate K^+^ demand (Horie et al., 2007). Besides Na^+^, OsHKT2;1 can also transport K^+^ when expressed in *Xenopus* oocytes (Jabnoune et al., 2009; Oomen et al., 2012), but K^+^ transport is not detected when it was expressed in yeast or in tobacco BY2 cells (Horie et al., 2001; Yao et al., 2010). In OsHKT2;1 expressing oocytes, the shifts in reversal potentials induced by K^+^ depended on the pre-treatment of oocytes. When the oocytes were pre-treated in low-Na^+^ (0.5 mM Na^+^) they showed smaller shifts that when pretreated with high-Na^+^ (96 mM Na^+^; Yao et al., 2010). Comparisons of Rb^+^ influx between WT and *oshkt2;1* roots did not reveal significant differences between these genotypes (Horie et al., 2007). Thus, the possible involvement of OsHKT2;1 in root K^+^ uptake remains to be verified. Another subfamily II HKT transporter, OsHKT2;2, is absent in the Nipponbare (japonica) cultivar but present in the indica cultivar Pokkali. The transporter obtained from the latter cultivar is permeable to both Na^+^ and K^+^ at low external concentrations when expressed in tobacco BY2 cells, yeast and *Xenopus* oocytes (Horie et al., 2001; Yao et al., 2010; Oomen et al., 2012). It is important to note that a natural chimera OsHKT2;2/1 present in the Nona Bokra (indica) cultivar maintains high-affinity K^+^ uptake even at high Na^+^ concentrations, something that is also observed for Pokkali OsHKT2;2 but not for Nipponbare OsHKT2;1 (Oomen et al., 2012). Despite the lack of data concerning *oshkt2;2* knock-out mutants, it is tempting to speculate that OsHKT2;2 contributes to both K^+^ and Na^+^ high-affinity uptake in rice roots. As for HAK transporters, two members, none of them from higher plants, have been shown to mediate high-affinity Na^+^ uptake: PpHAK13 from the moss *Physcomitrella patens* and YlHAK1 from the yeast *Yarrowia lipolytica* (Benito et al., 2012). PpHAK13, which belongs to cluster IV (Nieves-Cordones et al., 2016), transports Na^+^, but not K^+^, and high-affinity Na^+^ uptake is abolished in *pphak13* mutants plants which evidences that PpHAK13 forms the major pathway for Na^+^ entry at low external concentrations in *P. patens* plants. On the other hand, YlHAK1 is able to transport Na^+^ and K^+^ when expressed in yeast, but the latter cation is only transported when Na^+^ is not added to the experimental solution. High-affinity Na^+^ transporters from the HAK family in higher plants are still to be identified.

Concerning low-affinity Na^+^ uptake, it is generally accepted that Na^+^ can enter the plant through ion channels (Maathuis, 2014). Na^+^-permeable channels include glutamate-like receptors (GLRs; Davenport, 2002) and cyclic nucleotide gated channels (CNGCs; Assmann, 1995; Bolwell, 1995; Trewavas, 1997; Newton and Smith, 2004) and possibly other, non-identified, non-selective cation channels (NSCCs; Maathuis and Sanders, 1993; Tyerman et al., 1997; Demidchik and Tester, 2002; Essah et al., 2003). Voltage-independent NSCCs (VI-NSCC) may constitute the main class of NSCCs involved in Na^+^ entry since they are highly sensitive to Ca^2+^ as observed for Na^+^ uptake in roots (Demidchik and Maathuis, 2007). Moreover, VI-NSCC blockers such as quinine or lanthanides also inhibited root Na^+^ influx (Essah et al., 2003; Wang et al., 2006). Despite these encouraging observations linking VI-NSCCs and root Na^+^ uptake, the molecular identity of these channels remains obscure at present. Several HAK and HKT transporters, which are expressed in roots, are also permeable at millimolar Na^+^ concentrations (Santa-María et al., 1997; Horie et al., 2001; Takahashi et al., 2007; Mian et al., 2011; Oomen et al., 2012). It is worth to note that OsHKT2;1 and HvHKT2;1 contribute to Na^+^ uptake in the millimolar range but they are downregulated in the presence of salt stress or K^+^. Thus, when taking into account other experimental conditions and plant species, it remains unclear which other type/family of transport systems constitute the major pathway for low-affinity Na^+^ uptake. It is likely that there is a large redundancy between the aforementioned channels and transporters. Insights into the identification of the contributing transport proteins would be of extraordinary biotechnological value since low-affinity Na^+^ uptake allows for the massive entry of Na^+^ within the plant that gives rise to toxicity. Interestingly, the secondary messengers cyclic AMP and GMP affect Na^+^ influx. Studies on *Arabidopsis* seedlings (Maathuis and Sanders, 2001; Essah et al., 2003) and on pepper plants (Rubio et al., 2003) have shown that unidirectional Na^+^ influx is reduced by cGMP addition. CNGCs have a cyclic-nucleotide binding domain and their activity is modulated by cyclic-nucleotides (Gao et al., 2014, 2016). It can be expected that the cyclic-nucleotide regulation of Na^+^ fluxes occurs through direct cGMP (or cAMP) binding to this domain as it is the case of animal CNGCs (Craven and Zagotta, 2006).

Recently, it has been shown that salt stress triggers the formation of endocytic vesicles via a clathrin-independent mechanism (Baral et al., 2015). Such vesicles lead to the formation of vacuole-like structures that may help plants to better cope with salt stress. Endocytosis can modify the transporter complement of the plasma membrane (Sutter et al., 2007), thus affecting the Na^+^ uptake pathways. But more interestingly, the endocytic process involves bulk-flow entry into root cells that may transport Na^+^ ions from the apoplast to the vacuole-like structures. If this were true, it would constitute a parallel pathway for Na^+^ uptake, independent from that mediated by transmembrane proteins. Moreover, this direct Na^+^ transport into vacuoles would prevent Na^+^ accumulation in the cytosol which leads to cell toxicity. Interestingly, vesicle trafficking has been recently suggested to play a role in plant adaptation to salt stress (Garcia de la Garma et al., 2015).

## Concluding Remarks

K^+^ is an essential macronutrient for plants while Na^+^ may be beneficial or detrimental at low or high concentrations, respectively. Plant roots possess specific K^+^ transport systems that can function under a wide range of concentrations to secure K^+^ ions. The studies with knock-out mutants of the model plant *Arabidopsis* have led to the identification of two major pathways for K^+^ uptake: the high-affinity K^+^ transporter AtHAK5 and the inward-rectifying K^+^ channel AKT1. These systems operate at low (micromolar) and high (millimolar) external K^+^ concentrations, although an overlap in their operation is observed in the 10–200 μM K^+^ range. Different mechanisms that include transcriptional and post-transcriptional regulation modulate the activity of these two systems in response to K^+^ supply. Importantly, the CIPK23/CBL1 complex activates AtHAK5 as well as AKT1, pointing to its role as a central regulator of K^+^ nutrition. Homologs of AtHAK5 and AKT1 have been found in many plant species, and in some of them, paralog genes exist which suggest function redundancy. This precludes assigning a function by only using sequence homology or heterologous expression studies. The recent characterization of rice knock-out plants has shed light on this matter. OsHAK1, OsHAK5, and OsAKT1 seem to contribute to root K^+^ uptake as well as K^+^ release into xylem, and they probably play additional unknown functions in the shoot. However, they play non-redundant roles: (i) OsHAK1 is mainly involved in root K^+^ uptake at low concentrations, (ii) OsAKT1 mostly at high K^+^ concentrations, and (iii) OsHAK5 is more relevant for K^+^ translocation to the shoot. These systems may also play a role in salinity tolerance by maintaining the K^+^/Na^+^ homeostasis.

Regarding Na^+^ transport systems, the information is scarcer. High-affinity Na^+^ uptake and high-affinity Na^+^ transporters have been described in some species, but they are lacking in many others. The pathways for low-affinity Na^+^ uptake are not clearly identified yet, and several families of transporters contain members that could be good candidates. Recently, it has been described that salt stress induced a new endocytic pathway that is clathrin-independent, non-discriminatory in its choice of cargo, and that operates across all layers of the root. This new pathway may contribute to the bulk Na^+^ uptake and distribution across the root cells under saline stress conditions.

All of the above highlight the importance of characterizing the function of each transporter in each particular species. The studies with knock-out lines in *Arabidopsis* and rice evidence that the conclusions drawn in model species cannot be always fully extended to other, non-model species.

## Author Contributions

All authors listed, have made substantial, direct and intellectual contribution to the work, and approved it for publication.

## Conflict of Interest Statement

The authors declare that the research was conducted in the absence of any commercial or financial relationships that could be construed as a potential conflict of interest.
